# The Interplay of Public Intervention and Private Choices in Determining the Outcome of Vaccination Programmes

**DOI:** 10.1371/journal.pone.0045653

**Published:** 2012-10-01

**Authors:** Alberto d’Onofrio, Piero Manfredi, Piero Poletti

**Affiliations:** 1 Department of Experimental Oncology, European Institute of Oncology, Milano, Italy; 2 Department of Statistics and Applied Mathematics, University of Pisa, Pisa, Italy; 3 Predictive Models for Biomedicine and Environment, Fondazione Bruno Kessler, Trento, Italy; 4 DONDENA Centre for Research on Social Dynamics, Bocconi University, Milan, Italy; Northeastern University, United States of America

## Abstract

After a long period of stagnation, traditionally explained by the voluntary nature of the programme, a considerable increase in routine measles vaccine uptake has been recently observed in Italy after a set of public interventions aiming to promote MMR immunization, whilst retaining its voluntary aspect. To account for this take-off in coverage we propose a simple SIR transmission model with vaccination choice, where, unlike similar works, vaccinating behaviour spreads not only through the diffusion of “private” information spontaneously circulating among parents of children to be vaccinated, which we call *imitation*, but also through *public* information communicated by the public health authorities. We show that public intervention has a stabilising role which is able to reduce the strength of imitation-induced oscillations, to allow disease elimination, and to even make the disease-free equilibrium where everyone is vaccinated globally attractive. The available Italian data are used to evaluate the main behavioural parameters, showing that the proposed model seems to provide a much more plausible behavioural explanation of the observed take-off of uptake of vaccine against measles than models based on pure imitation alone.

## Introduction

The trend towards voluntary vaccination regimes, which many consider an irreversible process of modern industrialised countries, has given rise to substantial interest concerning the implications of vaccination free-riding for infection transmission and control. Vaccination free-riding arises when families decide not to vaccinate children, after a comparison of vaccination costs, given by the perceived risk of vaccine associated side effects, VSE, and benefits, given by the reduction in the risk of serious disease following infection. Free-riders in this case exploit the herd immunity created by others [1]–[4] to avoid VSE. Free-riding is a new form of “rational” opposition to vaccines [1]–[3], [5], which substantially differs from that observed in vaccination history, i.e. philosophical, religious, or conscientious objections [6]. A body of theoretical evidence has gradually accumulated, suggesting that free-riding makes it impossible to eliminate the infection [1]–[5], [7]–[11]. These results should be carefully taken into account in the design of immunization programmes.

Nonetheless the “elimination impossible” result requires some conditions with regards to agent behaviour on acquiring and handling information on perceived risks. For example in models based on evolutionary game-theoretic approaches to vaccinating behaviour [1], [4], [12], the information about the behaviour that is perceived as “better” spreads only through social contacts (which we label “imitation” ) between the parents of children eligible for vaccination, which is a seldom case in reality. In other words, even when a given vaccination is, formally speaking, voluntary, the public health system (PHS) will maintain the role of chief supplier of the relevant information on diseases and vaccines. It is mainly the information supplied by PHSs which avoids dramatic drops in vaccine uptake for diseases that are perceived as “not circulating” such as polio, or mitigates the impact of periods of vaccine scare. For example, in England and Wales, a system traditionally considered as voluntary, high measles coverages have been achieved by public incentives and subsidies targeting the groups of General Practitioners with whom a child is registered [13], [14].

Another interesting example is the recent dramatic increase in measles-mumps-rubella (MMR) vaccine uptake in Italy where some immunizations (diphtheria, poliomyelitis, tetanus, Viral Hepatitis B) were traditionally compulsory, while others (pertussis, measles, mumps, rubella) were only recommended. The major differences were that compulsory vaccinations were offered for free, necessary for school admission, with different vaccination schedules compared to those recommended [15]. The implication is that for compulsory immunizations uptake was always high, and geographically homogeneous, while it remained low, and inhomogeneous, for those recommended. In 1996 the diphtheria–tetanus–pertussis coverages in the Italian regions ranged between 90 and 99%, while for MMR it ranged between 25 and 80%, with a national average of 56% [16]. This dramatically low measles coverage, compared to the WHO target (95% first dose), made measles immunization one of the priorities of the PHS. The main actions taken were (a) the development, as from 1998 of a new nationwide immunization schedule unifying all pediatric immunizations, without distinction between compulsory and recommended immunization, (b) the free offer of MMR at the age of 12 months with other immunizations, (c) approval in 2003 of the National Plan for Measles and Congenital Rubella Elimination, allocating resources for further increasing first dose coverage, and for a national campaign targeting school-age children. Such measures allowed the first dose national coverage to increase to 77% in 2003, and then to 90% in 2008, with some regions above the WHO target, and with a marked decline in geographic inhomogeneity [15], [17]. All these suggest that the recent public health subsidies have put an end to the stagnation due to the long-standing voluntariness of the Italian MMR programme.

Based on previous arguments, we propose a new framework to predict the dynamic effects on vaccine uptake and transmission, of the interplay between private information, exchanged through inter-personal communication between parents of children eligible for vaccination during their social contacts, and public information, communicated by the Public Health authorities through the media and related channels. To this aim we amend the equation for the dynamics of the vaccinated proportion by coupling the imitation mechanism, where behaviour change spreads due to private information spontaneously communicated between individuals [1], [4], [12], with a mechanism not considered so far in the behavioural epidemiology literature, where behaviour change spreads due to the information about vaccination benefits provided by the public health system. In particular we assume that unlike private information, information conveyed by public health systems suggests a very small, possibly zero, perceived risk of vaccine side effects, and a large, possibly prevalence-independent, risk of disease. Communicating that vaccination provides a positive net benefit even if infection prevalence is actually close to zero, is the only strategy public health systems can adopt to avoid the coverage decline that might ensue if the perceived risk of disease is prevalence-dependent.

**Figure 1:**
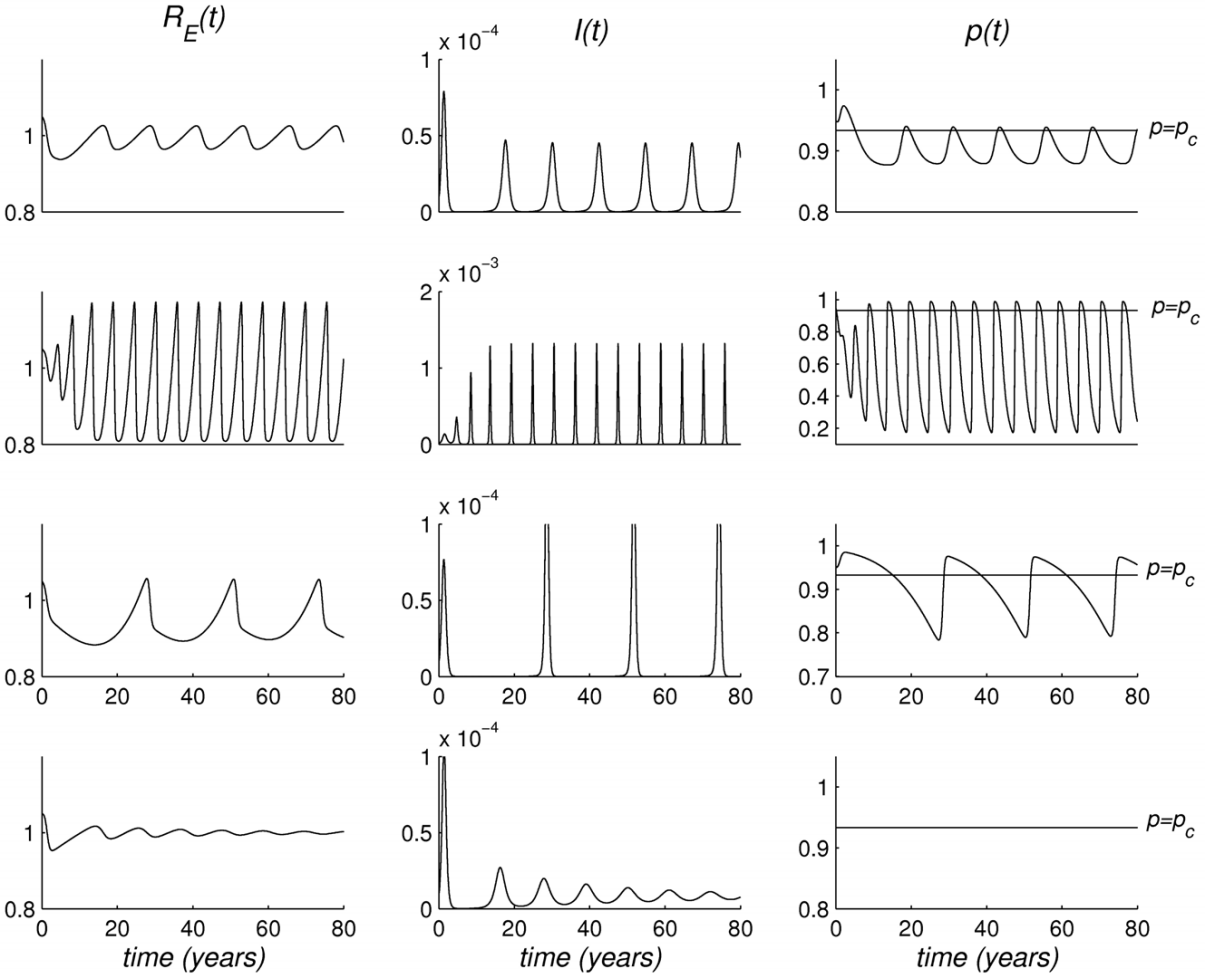
Dynamics of different models for vaccinating behaviour without and with public intervention. Top row: *G*-model with endemic coverage 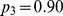 calibrated on Italian uptake of vaccine against measles in 2008; second row: *I*-model (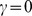) with endemic coverage 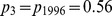 calibrated on Italian uptake of vaccine against measles in 1996; (c) third row: *I*-model with endemic coverage 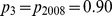 calibrated on Italian uptake of vaccine against measles in 2008; bottom row: the basic SIR model with constant coverage 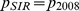. The figure reports time trends of the Effective Reproduction Number 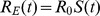 (left column), the infective fraction 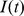 (centre), the vaccinated proportion 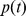 (right column). Other parameter values: 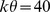, 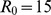, 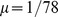 year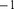, 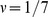
day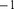. Initial conditions are: 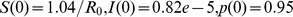.

Our main theoretical results show that a stabilising role can be played by public intervention, reducing the strength of “imitation” -induced oscillations, allowing disease elimination, and even making the disease-free “Pure Vaccinator Equilibrium” (i.e. where everyone is vaccinated, see [1]) globally attractive. Finally some illustrative scenarios on measles control are considered and a plausible explanation is proposed for the considerable changes observed in measles vaccine uptake recently observed in Italy. The previously reported Italian data on MMR uptake are used to roughly disentangle the relationships between main behavioural parameters. Our analysis shows that effective public interventions on information about vaccine benefits provides a more plausible behavioural explanation for the take-off in Italian MMR uptake than models considering only information spontaneously circulating in the population.

**Figure 2:**
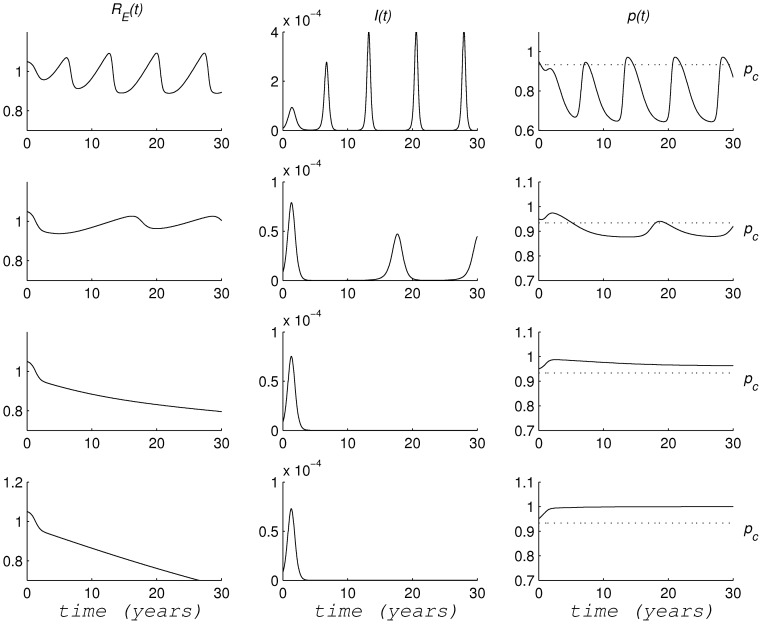
Dynamics of the *G*-model triggered by different levels of public intervention as represented through increasing values of 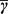. Top row: 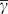 set to achieve 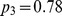 (Italian measles coverage in 2003); second row: 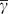 set to achieve 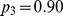 (Italian measles coverage in 2008); third row: 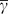 set to achieve elimination with 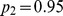 (WHO target for measles elimination); bottom row: 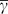 set to achieve the *PVE* 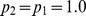. The figure reports time trends of the: Effective Reproduction Number 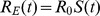 (left column), infective fraction 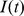 (centre), vaccinated proportion 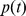 (right column). Other parameter values: 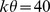, 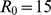, 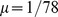
year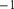, 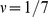
day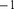. Initial conditions: 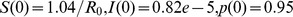.

**Figure 3:**
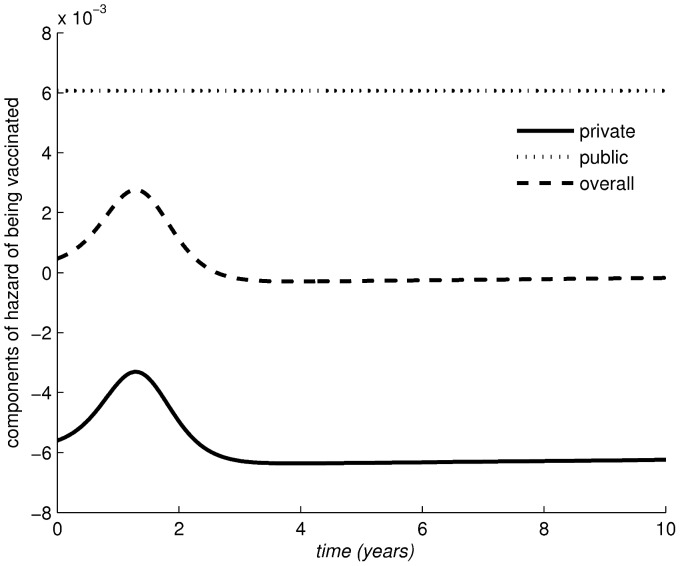
The elimination scenario occurring for 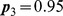: dynamics in the components of the hazard of getting vaccinated. Other parameter values: 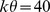, 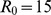, 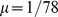 year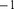, 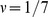 day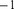.

## Methods

We consider a pediatric infectious disease, such as measles or mumps, which is controlled by a 100% effective vaccine ensuring lifelong immunity. Vaccination is administered at birth alone, i.e. we do not consider delayer strategies [3], [12]. Parents base their decisions to immunize their children or otherwise, on available information on the disease, e.g. on infection prevalence, or the incidence of cases of serious disease, and on the incidence of vaccine-associated side effects. Unlike previous work where the behaviour perceived as optimal is assumed to spread in the population through imitation alone [1], [4], i.e. via information exchanged essentially during social contacts through person-to-person interactions, we assume that behaviour can also spread through information provided by the PHS. Therefore we expand the dynamic equation for the vaccine uptake *p*(*t*) in [4], to include public information, as follows:
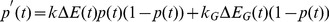
(1)


In (1) *p*(*t*) denotes the vaccinated proportion among newborn at time *t*, 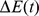 is the pay-off gain of vaccination that is perceived from information exchanged during person-to-person contacts, and *k* the related “imitation” coefficient, tuning the speed at which the pay-off perceived from person-to-person contacts creates new vaccinators. Similarly 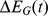 is the pay-off gain perceived from information spread by the PHS through its channels (e.g., the media, general practitioners, etc), and 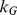 the “public acceptance” coefficient, tuning the speed at which new vaccinators are created by public information. Note that the public contribution, unlike the private one, does not include the 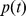 term, meaning that public communication affect those who did not vaccinate at a rate which is independent of their social activity. In simple words, the first part of eq. (1) models the change in vaccine uptake arising from information exchanged during social contacts between individuals who vaccinate and individuals not vaccinating, while the second part of (1) models the change in vaccine uptake arising from communications from the public health system to families who did not vaccinate.

**Figure 4:**
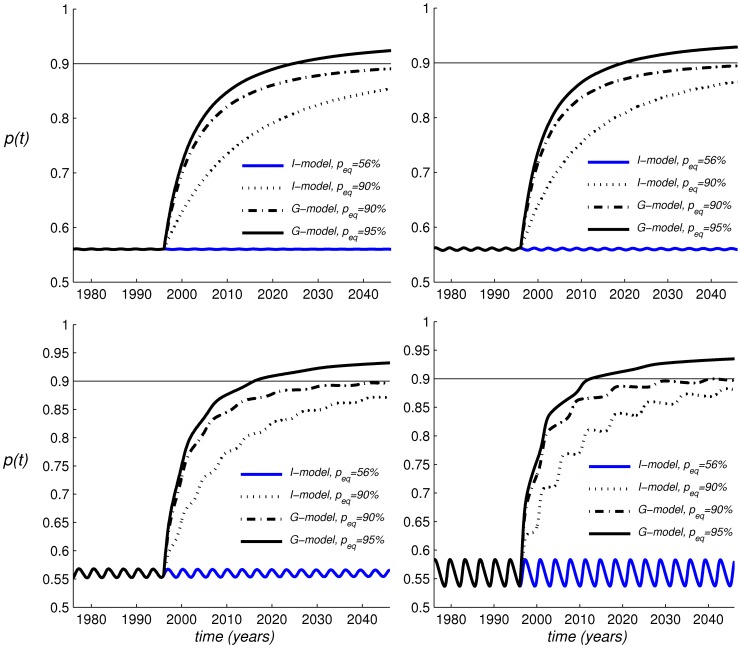
Dynamics of measles coverage in Italy during 1996–2040. Prediction of four different models: (a) the *I*-model with 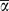 set at its pre-1996 level of 0.56; (b) the *I*-model, with 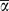 reduced in order to allow an equilibrium uptake of 90%; (c) the *G*-model with 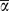 set at the pre-1996 level and 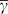 set in order to allow an equilibrium uptake of 90%; (d) the *G*-model with 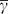 allowing an equilibrium uptake of 95%. The four models are initialised at 1996 after 20 years of steady dynamics implied by model (a). A flat line at the level 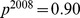 is also added for reference. The four panels are drawn for 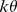 = 2.5 (North-West), 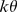 = 3.0 (North-East), 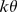 = 3.5 (South-West), 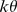 = 4.0 (South-East). All the four models are initialised from the endemic state of model (a): 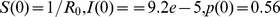.

Both perceived payoff gains in (1) are defined as the differences between the corresponding non-vaccinator costs, i.e. the perceived risk of suffering serious consequences from the disease, and the vaccinator costs, i.e. the perceived risk of suffering a vaccine-associated side-effect. Following [4] we define the payoff gain 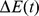 perceived from person-to-person contact as follows:
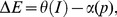
(2)where 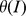 represents the perceived cost of the disease, taken as a function of the infection prevalence, and 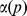 is the perceived cost of vaccination, i.e. the perceived cost of suffering a VSE. In particular 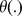 is an increasing function with 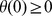, and 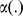 is an increasing function with 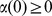. Among possible noteworthy subcases, we recall the linear forms for 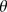 and 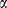. In particular the linear case 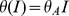 allows the perceived cost of the disease to be interpreted as the product of the perceived risk of infection, taken as a linear function 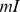 of infection prevalence [1], times the perceived conditional risk of disease given the occurrence of infection, taken as a constant 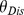. In this case 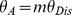. Similarly, 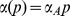 defines the perceived cost of suffering VSE as the product between the perceived risk of being immunized times the perceived conditional risk 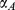 of suffering a vaccine side effect given the event of vaccination, according to the myopic mechanism illustrated in [4].

The pay-off gain 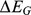 perceived from public information can be developed analogously. However, based on papers adopting the WHO position on vaccines and their use by the Italian PHS [18], we hypothesise a much simpler form. In fact, messages from the PHS aim to communicate that vaccines, besides being effective, are highly safe, with a very low, constant, risk of VSE, and that the risk of disease is prevalence-independent. The latter is obviously motivated by the need, for PHS, to avoid coverage decline during periods of falling prevalence (for example after a period of persistently high vaccine uptake). Therefore we assume that 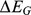 is simply constant. By defining 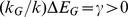, we end up with the following equation for the vaccinated proportion:
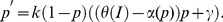
(3)


Notably, (3) extends the celebrated Bass model [19] for information diffusion by including pay-offs from both sources, private and public, of information spread. The parameter 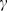 is the perceived payoff gain from adopting the public recommendation weighted by the ratio 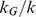 between the relative speeds of public and private information, which tunes the strength of public vs private acceptance. Therefore 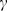 summarises the effectiveness of the public actions (information, education, availability of vaccination infrastructures, including monetary subsidies to vaccination staff) in affecting perceptions on vaccines and disease.

By putting (3) into the standard homogeneously mixing Susceptible-Infective-Recovered (SIR) model with vaccination choices [1], [4] we get:
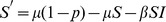
(4)

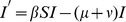
(5)

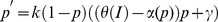
(6)where the two further state variables *S*, *I*, respectively denote the fractions of susceptible and infective individuals, 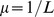 denotes the birth and death rates, where *L* is the life expectancy at birth, 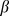 the transmission rate, and 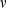 the rate of recovery from infection. For the sake of brevity from now on we will refer to model (4)−(5)−(6) as the *G*-model, and will label as the *I*-model the “pure imitation” model used in [4], which stems from the *G*-model by setting 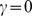.

Let 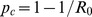 represent the critical elimination coverage in the *G*- and *I*- models, where 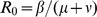 is the Basic Reproduction Number, representing the number of secondary infections caused by an index case in a wholly susceptible population. In the next section we state the main results elucidating the effects of public intervention on the steady states of the *G*-model, and the related stability (mathematical details available as supporting text).

## Results

### Control of Re-emerging Infections

A first interesting result concerns the situation where zero incidence has been achieved, e.g. by a vaccination campaign. In this case 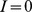 implies 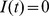 at all future times, and the model collapses into the decoupled 2-dimensional system:
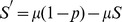


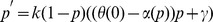



In this case the condition 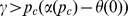 ensures that the vaccine uptake eventually achieves an equilibrium in excess of the critical coverage 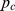. The quantity 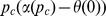 therefore represents the “maintenance threshold” of public effort, above which the community is permanently protected against external reintroduction of infection. Obviously this threshold is higher if 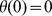, i.e. when the perception of a risk of external reintroduction only comes from the PHS.

### Public Intervention, Equilibria and Stability

Unlike [1] the *G*-model does not allow Pure Non Vaccinator steady state where none vaccinates. This is a straightforward consequence of the presence of public intervention. The model has a Pure Vaccinator Equilibrium [1], i.e. a disease-free state 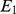 where everyone is vaccinated (we will also denote it as PVE):
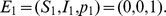



It may be shown that high values of 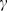, i.e. 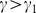, where 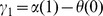, ensure the global attractivity of the PVE. Conversely, when 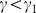 the 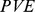 is unstable (see the Supporting Information). Moreover the system admits a second disease-free state:
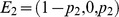
where 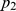 is the unique solution of the equation:



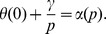



This disease-free state bifurcates from the 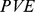 when 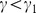. It is possible to show (see the Supporting Information) the existence of a threshold value 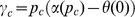, such that if 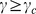, then the equilibrium uptake 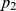 exceeds the critical coverage 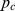 and 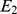 is globally attractive. Note in particular that 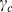 represents the above “maintenance threshold”. Further, if 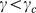 (i.e., 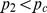) then 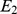 is unstable. When 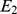 becomes unstable a unique (and epidemiologically meaningful) endemic state 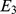 appears:
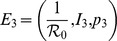
by a transcritical bifurcation at 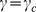. The endemic state 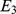 is always locally stable when a suitable function 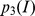, tuning the intensity of the behavioural response to changing perceptions, is sufficiently flat at 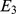, i.e. for 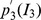 smaller than a suitable value *W*. Conversely when the function 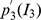 becomes sufficiently steep, i.e. for 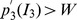, then 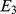 will be unstable in a suitable intermediate window of values of the quantity 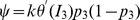 which tunes the average delay with which changes in perceived risks affect vaccine uptake in the endemic state [4]. The ensuing dynamics will then be oscillatory in the sense of Yabucovich [20], [21] general sustained oscillations, whose nature - periodic, quasi-periodic or chaotic - cannot be specified a priori. Nonetheless, our numerical simulations suggest that oscillations mostly have the form of stable limit cycles. These results suggest that when the role of public information is mild then the model qualitatively behaves like the model described in [4], where the driving force of oscillations was the agents’ reaction to epochs of changing prevalence under a suitable pattern of delay in the spread of the behaviour perceived as optimal during social contacts.

### Noteworthy Consequences of Public Intervention

The previous results show the interplay of the two information providers on vaccination payoffs, i.e. the public and private sectors, in affecting the dynamics of infection and vaccine uptake. Since the locations of both 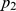 and 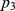 are continuous increasing functions of gamma, this interplay can be summarised by highlighting the role of the 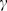 parameter in tuning the intensity of the public effort:

- For very strong levels of public effort (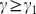) only the Pure Vaccinator Equilibrium exists and it is globally asymptotically stable (GAS).
- For intermediate levels of public effort (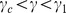) the PVE is unstable, but the disease-free state 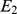 exists and it is GAS (since 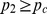).
- For low levels of public effort (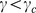) the elimination equilibrium 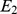 exists but is unstable (because in this case 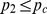), and the endemic state 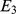 exists. Note that 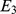 is locally asymptotically stable (LAS) in some cases, but might also become unstable.

In a control perspective, if the infection is endemic and the public intervention is mild or absent, it is possible to increase the equilibrium coverage by increasing the public effort in providing information about the benefits of vaccination. Suitable further increases in public effort can allow the equilibrium vaccine uptake to expand until the endemic state 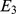 disappears by exchanging its stability with the disease-free equilibrium 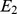, thus achieving elimination. Further increases in 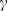 yield further increases in vaccine uptake, until 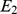 collapses into the PVE (i.e., 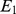). In particular, values of 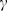 such that 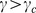 modulate the speed of elimination.

Recalling that 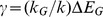, we can give the previous conditions a more meaningful interpretation in terms of payoffs. For example the condition for the global stability of the PVE may be written as 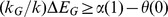, i.e. as:
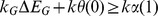



This relationship states that if the overall perceived risk of disease when 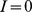 exceeds the overall perceived risk of VSE when everyone vaccinates (i.e. 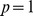), then the PVE is GAS. Note that the previous condition depends not only on the magnitude of perceived quantities, but also on the relative time scales of spread of private and public information.

Overall, our results show that public intervention (a) always allows the establishment of some positive level of vaccine uptake because, unlike the 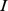-model [1], [4], it prevents the existence of pre-vaccination steady states; (b) allows a plausible mechanism for the elimination equilibrium 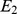 to be globally attractive; (c) allows, when very strong, the PVE to become globally attractive.

Finally, in the noteworthy case where the risk of VSE perceived from inter-human communication is constant, we obtain, as regards the existence and stability of the equilibria, the same patterns as in the case of non-constant 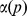.

### Interplay of Public and Private Information, and Measles Control

Given the impossibility of fitting behavioural parameters due to the paucity of data, we attempted at least to disentangle the relative role of private vs public information by using the few Italian data on measles coverage reported in the introduction. We hypothesise that: (a) the “low” uptake of vaccine against measles (56%) observed in 1996 reflected the steady state of a fully voluntary immunization program, based on the *I*-model; (b) the sharp increase in uptake observed during 1996–2008 mirrors, at least crudely, a new steady state situation, implied by the initiation of a public programme which rapidly raised 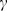 from zero up to a positive value, on the assumption that the imitation-related parameters remained constant during the same period. As for the demo-epidemiological parameters, the life expectancy 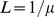 is set at 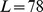 years, which is representative of Italian mortality at the beginning of 2000, while the basic reproduction number and the recovery rate for measles are set at 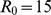, implying 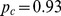
3, and at 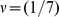 day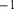 respectively. We also assume, for the sake of simplicity, that perceived risks from private information are linear functions: 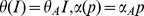, 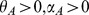.

In this case the condition for the global stability of the PVE becomes simply: 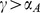. For 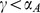 the disease-free equilibrium 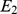 appears, with 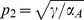, and is GAS for 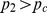, that is for 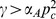. On the other hand for 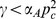, 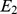 becomes unstable and the endemic state 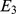 appears. By setting 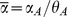, 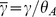, we have: 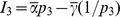, where 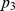 is the positive solution of the following second order algebraic equation in 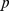


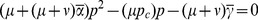
(7)


### Determining Model Parameters from Italian uptake Data

Under the previous assumptions we can determine the relationships between the main behavioural parameters as follows. The routine uptake of vaccine against measles 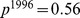 observed at 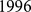 is taken as the equilibrium uptake of an underlying *I*-model (i.e., by assuming 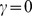), given by
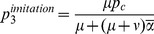
(8)


By equating 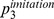 to 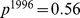 we can solve (8) for 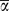, getting 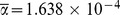, i.e. 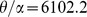. This large disproportion between 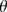 and 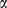 is a consequence of the hypothesis that the perceived risk of infection is prevalence-dependent [4]. Note that achieving the value 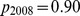 of routine uptake of vaccine against measles observed in 2008 as equilibrium coverage would require, under imitation dynamics only, a 20-fold decline in 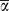, up to 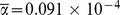. This large drop suggests that the marked increase in vaccine uptake observed in Italy in such a short period of time is unlikely to have been achieved by changes in costs perceived during spontaneous contacts between individuals alone.

Next, let us determine 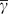 from the equation 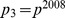 on the assumption that 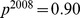 represents the endemic uptake 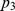 of the *G*-model, given by (7), and that 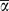 remained unaltered during 1996–2008. This yields 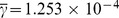. This implies 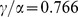, and hence that 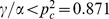, i.e. that the system is in its endemicity region. In order to achieve elimination it would be required that 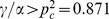, as already stated.

### Simulations

Using the information on observed coverage at only two time points we have been able to determine only the ratios between the behavioural parameters 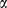, 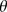, 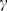. Therefore these parameter values are compatible with a wide variety of dynamic endemic regimes, depending on the product 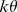, as is clear by reparametrizing the eq. (6) for the dynamics of the vaccinated proportion as follows:
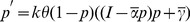
(9)


In particular for the computed values of 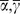 the endemic state 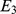 can be destabilised, and the corresponding Hopf points are 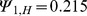, and 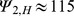. By recalling that in this case 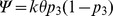, and that 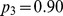, we therefore have that 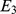 is unstable when the product 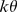 ranges between 2.38 and 1277 about.

To investigate the involved dynamic regimes, we simulate a slightly modified version of the *G*-model, including a small constant transfer *Imm* per unit of time from the susceptible to the infective state [1], [4]. Note that in the absence of this immigration term the model would allow an oscillatory regime with values of 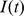 close to zero for which a stochastic model may be more appropriate. *Imm* is set at one infective individual per week in a population of 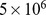 individuals. Obviously this prevents “full elimination” of the infection, so that when later on we speak of elimination we mean “disregarding importation” of cases.

Fig. 1 compares, over a time horizon of 80 years, the predicted dynamics of the *G*-model with endemic vaccine uptake equal to the value observed in Italy in 2008 (i.e. 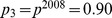) after the big public effort to increase measles immunization (Fig. 1, top row) and a value of 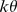 in the oscillations region (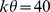), with those of two underlying *I*-models for the same values of 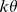: the *I*-model with endemic coverage 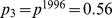 as in the period when measles immunization was only recommended (Fig. 1, second row), and the *I*-model with equilibrium coverage 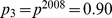 (Fig. 1, third row). Dynamics of the basic SIR model with constant coverage 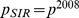 are also reported (Fig. 1, bottom row). While the basic SIR model shows the traditional damped oscillations, the *I*-models (Fig. 1, second and third row) swiftly achieve a stable limit cycle, characterised by wide oscillations. In particular for low equilibrium uptake (Fig. 1, second row) the inter-epidemic period is slightly below five years due to the rapid susceptible replenishment, while in the case of the larger equilibrium uptake (Fig. 1, third row) the inter-epidemic period exceeds 20 years, as a consequence of the high average coverage achieved. When public intervention is included (Fig. 1, top row), the system still lands on a limit cycle, due to the high speed of information diffusion. However, the period of this oscillation is about 10 years, i.e. much shorter than the corresponding model with imitation dynamics only (Fig. 1, third row) and close to the figure of the basic SIR model. In particular public intervention has a strong stabilising effect on the oscillations of vaccine uptake, which are now confined in the region between 87 and 94%. Public intervention also plays a stabilising role in sharply reducing the amplitude of the instability window of 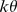 compared to *I*-model [4].

Fig. 2 illustrates the impact (over a time span of 30 years) of different levels of public intervention as represented through increasing values of 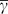, aiming to achieve (other things being equal) the following targets of vaccine uptake: (top row) endemic equilibrium coverage given by: 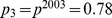, i.e. the measles coverage recorded in Italy in 2003 after the first big wave of public intervention, (second row) 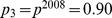, (third row) elimination coverage with 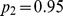, which is the WHO target for measles elimination, (bottom row) elimination with everyone vaccinating (the 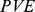) 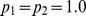. The first two scenarios predict disease persistence, and confirm the stabilising role played by public information. The last two scenarios yield elimination and show the interesting fact that though public intervention is unable to avoid a large initial epidemic due to the large initial susceptible fraction, it is subsequently able to avoid the drop in uptake that would unavoidably occur in an *I*-model as a consequence of the large number of vaccines administered. The large initial epidemic creates a phase where the perceived risk of disease is high thereby also speeding up the private component of vaccination, which in this case works synergically with the public one in accelerating disease elimination. This effect is clarified for the elimination scenario 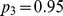 by separating (Fig. 3) the hazard 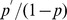 of becoming a vaccinator at any time, into its two information components, i.e. imitation and public. The public component is by definition constant. By contrast the “private” one has a hump during the big initial epidemic, due to the peak in the perceived risk of infection, which rapidly increases the hazard of getting vaccinated, but then drops as the epidemics is over and the number of VSEs increases, and finally stabilises. Note that the imitation component is always negative here, being “calibrated” on data predicting an equilibrium state with low coverage. However, the high level of the public component however is capable of balancing the negative role of imitation, and rapidly achieving elimination with a peak in the speed at which individuals are vaccinated during the initial epidemics. Here a possibly important public health message emerges, i.e. that large epidemics triggered by periods of vaccine distrust might represent opportunities for elimination provided the public health system can exploit it timely, by pumping into the system appropriate resources able to synergically exploit the hump in the perceived risk of disease due to the just-occurred epidemics.

### Public Intervention Explains the Take-off of MMR Uptake in Italy

Having used only two data points only allowed to identify the ratios (i.e., the quantities 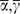) between main behavioural parameters. Since nothing could be inferred about 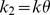, whether the *G*-model explains the growth in uptake of vaccine against measles recently observed in Italy remains an open question. To tackle it we extensively investigated the response of model behaviour to changes in 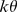, conditionally on the values determined for 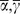. Our purpose here was to identify combinations of 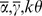 compliant with the patterns of vaccine uptake observed in Italy during 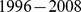 and compare predictions provided by the proposed *G*-model with those based on the *I*-model. Fig. 4 reports, for four increasing values of 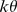, the behaviour of vaccine uptake 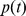 in four alternative models: (a) the *I*-model with 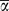 set to allow an endemic vaccine uptake 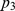 equal to the level of 0.56 observed in Italy prior to 1996; (b) the *I*-model, with 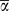 reduced to allow an endemic vaccine uptake of 90% as observed in Italy in 2008; (c) the *G*-model with 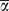 set at the pre-1996 level and 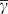 set to allow an equilibrium uptake of 90%; (d) the *G*-model with 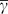 allowing an equilibrium uptake of 95%, i.e. the first dose target of the National Elimination Plan for measles. The four models are all initialised at 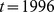 after a 20-year stagnation period, since we hypothesised that prior to 1996 vaccine uptake was at the steady state of a *I*-model with coverage of 56%. A flat line at the level 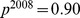 is also added for comparison. Model (a) continues to follow its equilibrium pattern (stationary or oscillatory). On the other hand the other models predict a sharp increase in vaccine uptake (note the increase is monotonic for values of 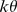 below a threshold, and oscillatory thereafter). Nonetheless in the *G*-model vaccine uptake grows towards its steady state much faster than the *I*-model regardless of the chosen value of 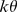, although the imitation model is now evolving under an implausibly large value of 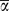, which is 20-fold lower compared to the pre-1996 period, thereby implying an implausible decrease in relative (perceived) risk of vaccine side effects compared to the past. Thus the *G*-model seems to account for the observed growth in MMR uptake much better than the *I*-model. In particular, the *G*-model with equilibrium uptake of 95% allows - for some values of 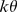 - to closely reproduce the observed growth of measles vaccine uptake during 1996–2008. We also note that the same result can only be approached in the *I*-model by implausibly large 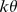 values yielding, huge, unreasonable oscillations.

## Discussion

We expanded the SIR model for vaccine preventable infections and vaccination choice based upon an imitation dynamics [1], [4], to take into account the role of the public health system as the main provider of information on diseases and vaccines. The ensuing mathematical model suggests that public intervention can offset the pessimistic conclusions based on models with imitation dynamics alone [1], [4]. In particular the intervention of the public health system is shown to always play a stabilising role able to reduce the strength of imitation-induced oscillations in vaccine uptake, remove the “elimination impossible” result, and even make, when sufficiently strong, the disease-free equilibrium where everyone vaccinate globally attractive.

From the empirical point of view, due to the lack of appropriate data it is still a challenging task to validate current models explicitly including behavioural changes. Nonetheless, the analysis proposed here, based on scenarios compliant with current observations represents a further step in using simple models to identify important mechanisms underlying non-pharmaceutical interventions. Indeed, given the difficulty of directly separating the role of private vs public information in determining the observed vaccine uptake, models like the present one can profitably used to identify behavioural parameters indirectly. In this first effort we attempted a rough evaluation of the main parameters related to vaccination behaviour, hypothesising that the few available data on the recent history of uptake of vaccine against measles in Italy represent a switch between two distinct equilibrium regimes. The ensuing scenarios suggest that our model seems to be able to offer a much more plausible behaviour-based explanation of the rapid increase in measles vaccine uptake observed in Italy compared to the “pure imitation” model. Though it could be stated that the observed uptake growth could be predicted similarly well by a simpler model without behavioural components but just including a time-increasing exogenous uptake, the present model has the advantage of incorporating a well-posited behavioural explanation. Overall we believe that accounting for public information, does not simply represent the inclusion of a further parameter into the imitation model, but represents instead a parsimonious way to account for a necessary component of current infection dynamics in highly vaccinated populations. In particular our results suggest that public intervention might be a critical resource in order to ensure a rapid increase in vaccine uptake in situations where individuals choices have caused policy stagnation.

A critical point of previous results is that the effectiveness of the public health actions can not be taken as linear, as postulated here, whatever the level of the vaccine uptake. More reasonably it will be nonlinear, possibly saturating at very high vaccine uptake, mirroring the difficulties in capturing “marginal” individuals. This issue could not be tackled with the few data available, but is worth considering for future research.

More general criticism might concern the role of simple models, like ours, due to their lack of realism over a variety of important dimensions. Therefore it would be important to test the robustness of their predictions with respect to both behavioural and epidemiological refinements, in order to assess the role of different assumptions on alternative decision mechanisms [22], [23], of age heterogeneities and network structures in infection transmission [24], of the interplay between information and infection transmission networks [23], [25]–[29]. For example, as for the modeling of vaccination choices, an alternative approach to the present one could be to divide decision-makers regarding vaccination into two groups: one consisting of those who are influenced by other individuals; the other consisting of those who are influenced by public interventions. Other critical points lie, although it is nowadays definitively acknowledged that behaviour matters [30], in the persistent lack of appropriate direct data on behaviour against which to ground the predictions provided by the rapidly increasing interest for the subject [1]–[4], [8], [10], [11], [22]–[29], [31]–[37]. Here the challenge lies in the appropriate design of field work in order to estimate behavioural parameters reliably and hence allow behavioural-epidemiological models to make the leap from their current role of elegant theoretical tools to that of useful policy-supporting tools. For example recently an Italian region [38] has, for the first time, removed mandatoriness for all immunizations, including those (polio, diphtheria, HBV) where coverage was high. The same decision might be adopted in other regions, where the issue is currently being debated. Gaining insights into the mechanisms underling risk perception, and consequent behavioural responses would appear the only way to obtain valuable tools for providing robust predictions on the outcome of such critical processes.

## Supporting Information

Supporting Information S1 **In this appendix proofs of main mathematical results are presented.**
(PDF)
