# Supplementary material for: The Interplay of Public Intervention and Private Choices in Determining the Outcome of Vaccination Programmes
Source: PLoS One. 2012 Oct 1;7(10):e45653. doi: 10.1371/journal.pone.0045653 (PMC3462214; doi:10.1371/journal.pone.0045653)
Supplement: Supporting Information S1 — In this appendix proofs of main mathematical results are presented. (PDF) [file pone.0045653.s001.pdf]

Supporting materials to manuscript: The interplay of public intervention and private choices in determining the outcome of vaccination programmes

Alberto d'Onofrio[1], Piero Manfredi[2] *, and Piero Poletti[3]

June 30, 2012

[1] Department of Experimental Oncology, European Institute of Oncology, Via Ripamonti 435, Milano, Italy
EMAIL: alberto.donofrio@ifom-ieo-campus.it

[2] Department of Statistics and Applied Mathematics, University of Pisa. Via Ridolfi 10, Pisa, ITALY.
EMAIL: manfredi@ec.unipi.it

[3] Predictive models for Biomedicine and Environment, Fondazione Bruno Kessler, Via Sommarive 18, Trento, Italy
EMAIL: poletti@fbk.eu

1 Proofs of main mathematical results

1.1 Global stability of PVE

Let us assume that $\gamma > \gamma_1$, where $\gamma_1 = \alpha(1) - \theta(0)$. Recalling that both $\alpha(p)$ and $\theta_0(I)$ are increasing functions, the following differential inequality holds:

$$p' > k\left(\gamma + p(\theta(0) - \alpha(1))\right)(1-p) \tag{1}$$

implying that :

$$\liminf_{t\to+\infty} p(t) \geq Min\left(1, \frac{\gamma}{\alpha(1) - \theta(0)}\right). \tag{2}$$

As a consequence, if $\gamma > \gamma_1$ holds then $\liminf_{t\to+\infty} p(t) \geq 1$, thereby showing that the PVE is GAS. Conversely, if $\gamma > \gamma_1$ does not hold, the instability of PVE easily follows by linearising the dynamic equation for p at the PVE.

1.2 Global stability of E_2

Assume now that $\gamma_c < \gamma < \alpha(1) - \theta(0)$. Consider the following differential inequality

$$p' \geq kp(1-p)\left(\theta(0) + \frac{\gamma}{p} - \alpha(p)\right) \tag{3}$$

it follows that

$$\liminf_{t\to+\infty} p(t) = p_2.$$

*Corresponding Author

Moreover, the above minimum limit implies that for large times :

$$S' \leq \mu(1 - p_2 - S)$$

and in turn, that:

$$I' \leq \beta I(p_c - p_2).$$

Now, note that elementary analysis implies that if $\gamma \geq \gamma_c$ then $p_2 > p_c$. Thus the global stability of E_2 immediately follows. Conversely, if $\gamma \leq \gamma_c$ then $p_2 < p_c$, and the instability of E_2 follows from the linearized equation for the infective fraction:

$$i' = \beta i(p_c - p_2).$$

1.3 Existence and local stability of the endemic state E_3, and Yabucovitch property

Assume now that $\gamma \leq \gamma_c$, which makes the Disease Free state E_2 unstable, and let us show that the unique (and epidemiologically meaningful) endemic state E_3 appears:

$$E_3 = \left(\frac{1}{\mathcal{R}_0}, I_3, p_3(I_3)\right)$$

where p_3 is a function of I determined by the equation:

$$\theta(I) + \frac{\gamma}{p} = \alpha(p), \tag{4}$$

which follows from setting $p' = 0$.
Note first that $p_3(.)$ is increasing in I, with $p_3(0) = p_2$, and $p_2 \leq p_3(I) \leq 1$. Moreover, by applying the implicit function theorem to (4) one yields:

$$p'(I) = \frac{\theta'(I)p^2}{\gamma + \alpha'(p)p^2} > 0. \tag{5}$$

Finally, I_3 is determined from the equation:

$$p_3(I) = p_c - \left(1 + \frac{\nu}{\mu}\right) I \tag{6}$$

which has a unique meaningful solution provided that $p_2 < p_c$, and no solutions otherwise.
As for the stability of E_3 the following proposition holds

Proposition 1.1 *If:*

$$p_3'(I_3) < W, \tag{7}$$

where:

$$W = \frac{\mu + \beta I_3}{\mu \beta I_3}\left(\mu + \beta I_3 + 2\sqrt{\beta I_3(\mu + \nu)}\right),$$

then the endemic equilibrium E_3 is locally asymptotically stable.
If

$$p_3'(I_3) > W \tag{8}$$

two values: $u_1 =$, $u_2 =$ exist, such that:
i)*If*

$$k\theta'(I_3)p_3(1 - p_3) \in (0, u_1) \cup (u_2, +\infty) \tag{9}$$

then E_3 is LAS;
ii)*If*

$$k\theta'(I_3)p_3(1 - p_3) \in (u_1, u_2) \tag{10}$$

then E_3 is unstable and the orbits $x(t) = (S(t), I(t), p(t))$ are oscillatory in the sense of Yabucovich [1, 2], i.e. for $j = 1, 2, 3$ it holds that:

$$minlim_{t \to +\infty} x_j(t) < maxlim_{t \to +\infty} x_j(t).$$

Proof To prove the proposition, let us define the quantities $\Psi = k\theta'(I_3)p_3(1-p_3)$ and $A = (p_3'(I_3))^{-1}$. The Jacobian matrix at E_3:

$$J3 = \begin{bmatrix} -(\mu+\beta I_3) & (\mu+\nu) & -\mu \\ \beta I_3 & 0 & 0 \\ 0 & \Psi & -A\Psi \end{bmatrix} \tag{11}$$

yields the characteristic polynomial $\lambda^3 + b_2\lambda^2 + b_1\lambda + b_0$ with coefficients:

$$b_2 = A\Psi + q_2$$

$$b_1 = Aq_2\Psi + q_1$$

$$b_0 = Aq_1(1+r_0)\Psi$$

where:

$$q_2 = \mu + \beta I_3$$

$$q_1 = \beta I_3(\mu+\nu)$$

$$r_0 = \frac{\mu}{\mu+\nu}\frac{1}{A}$$

The positivity of the coefficients of the characteristic polynomial rules out, by Descartes theorem, the possibility of real positive eigenvalues, so that stability losses of the endemic state can only occur via Hopf bifurcations. The Routh-Hurwitz condition $b_1b_2 - b_0 > 0$ reads:

$$q_2A^2\Psi^2 + \left(q_2^2 - q_1r_0\right)A\Psi + q_1q_2 > 0 \tag{12}$$

Thus it is easy to see that if:

$$r_0 < \frac{q_2}{q_1}\left(q_2 + 2\sqrt{q_1}\right),$$

i.e. if $p_3'(I_3) < W$ then E_3 is LAS. On the other hand, if $p_3'(I_3) > W$ then the equation associated to (12) has two solutions, u_1 and u_2, so that if (9) holds E_3 is LAS, whereas if (10) holds then E_3 is unstable. As regards Yabucovitch oscillations, note that: *i)* the bounded set

$$A = \{(S,I,p) \in R_+ | 0 \le S + I \le 1 - p_2(\gamma), p_2(\gamma) \le p \le 1\}$$

is positively invariant and attractive; *ii)* E_3 is unstable, and E_2, which is in the boundary of A, has as the stable manifold the plane $(\sigma, 0, \pi)$ with $(\sigma,\pi) \in [0,1]^2$ (absence of initial infectious subjects) to which E_3 does not belong, excluding heterocline orbits. Thus we may apply the Yabucovitch theorem [1, 2]$\diamondsuit$

References

[1] Efimov D.V. and Fradkov A.L. 2009 Oscillatority of nonlinear systems with static feedback. *Siam J. Contr. Optim.* **48** 618–640.

[2] Efimov D.V. and Fradkov A.L. 2008 Yakubovich's oscillatority of circadian oscillations models, *Mathematical Biosciences*, **216**, 187-191
